# Supplementary material for: Chromothripsis during telomere crisis is independent of NHEJ, and consistent with a replicative origin
Source: Genome Res. 2019 May;29(5):737–49. doi: 10.1101/gr.240705.118 (PMC6499312; doi:10.1101/gr.240705.118)
Supplement: Supplemental Material [file supp_gr.240705.118_Supplemental_file_1.zip › contigs/annotated_contigs/DB116/contig.2.DB116_length_564_mean_cov_8.51063829787.docx]

**DB116_length_564_mean_cov_8.51063829787**

```
AAATTCCCCATTGCCCTGACTTCCCTGGTTAAGAGTTCATAACATTGAGGTCTGAAATTTTGCTTCATTGCAGAATCAGAATATCTGCA
 >chr4:45157741-45157904 - E=2e-86
ATCTGCAATGTTTGTTTACCTTTATATAACCCAATCTTCTCTCTGTAGTAGATAAGAACTTCCTATAGAGAAAA|TCCATA|GCACACT
                                                                                 >chr4:
GCTATGTTTCCCAGCCTCTTTTGCATTTGACAGGGAGGATGGGCTTGAGTTCCACCCAATAGAATATGAGAAAAAATGATAAGAATTAT
45155401-45155796 - E=4e-223
TTCCAGGTCTTTGCTTAAAAAATAATTTCCTGGCCAGGTGACTGAGCAAGACTCATCTCCAATAAATAAATAAATAAATAAAAATAATA

ATTTCCCATGTGATTTTCTGCACTTTTCCCTTTTTGGTTGGCTTGGTGAAAATGAACACAATAATCTACATCATGGTAGAACCACAAGA

TAAAAAGAAGCATCCCGGAACATGACTTAGAGGAAAACCACTTGCCATAAAGAATACTCAGTTTGGACTTTAGATGAGTAAAATATATA

TTTCTGTTTGTTCGCTTGCTTGTTTGTGACAT
```
